# Lesser omental hernia through a defect in the posterior layer of the lesser omentum

**DOI:** 10.1186/s40792-023-01651-6

**Published:** 2023-05-04

**Authors:** Hirotaka Shibuya, Keita Sato, Yosuke Yamauchi, Yoshihisa Tamura, Koji Takahashi, Yasushi Asari

**Affiliations:** 1 Department of Surgery, Japanese Red Cross Ise Hospital, 1-471-2 Funae, Ise, Mie 516-8512 Japan; 2 Department of Emergency and Critical Care Medicine, Kitasato University School of Medicine, Sagamihara, Kanagawa Japan

**Keywords:** Hernia, Lesser omentum, Posterior layer, Computed tomography

## Abstract

**Background:**

In previously reported cases of lesser omental hernia, a rare clinical presentation, the herniated intestinal tract was passing through both peritoneal layers of the lesser omentum to herniate into the peritoneal cavity or bursa omentalis. Here we present a very rare case of lesser omentum hernia, where the transverse colon entered through only the posterior layer of the lesser omentum to form a hernia between the anterior and posterior layers.

**Case presentation:**

A 43-year-old man was admitted to the emergency department with acute abdominal pain. Plain abdominal computed tomography (CT) revealed a change in the caliber of the transverse colon between the stomach and pancreas, forming a closed loop on the cephaloventral side of the stomach. On contrast-enhanced CT images, vessels were observed in the contrast-enhanced lesser omentum surrounding the herniated intestine. The patient was diagnosed with a lesser omental hernia and underwent laparoscopic surgery. Intraoperatively, the transverse colon was covered by the anterior layer of the lesser omentum, and a defect was found in the posterior layer of the lesser omentum on the dorsal side of the stomach. A 2-cm incision was made in the posterior layer of the lesser omentum to widen the small defect. The herniated intestinal section was removed from the hernia sac, and the transverse colon was retained unresected. The postoperative course was uneventful.

**Conclusions:**

As illustrated in this first case of a lesser omental hernia forming between the anterior and posterior layers, characteristic CT findings may play an active role in the diagnosis of this rare presentation.

## Background

Lesser omental hernia is an extremely rare form of internal hernia [1]. The lesser omentum is a double-layered peritoneal structure [2]. In previous reports of lesser omental hernias, the herniated intestinal tract was reported to pass through both peritoneal layers of the lesser omentum to herniate into the peritoneal cavity or bursa omentalis [1, 3–11]. No study to date has reported the passage of the intestinal tract through only one of the peritoneal layers of the lesser omentum to form a hernia between the two peritoneal layers of the lesser omentum.

In this study, we report the first case of a patient in whom the transverse colon entered through only the posterior layer to herniate between the anterior and posterior layers of the lesser omentum.

## Case presentation

**Fig. 1:**
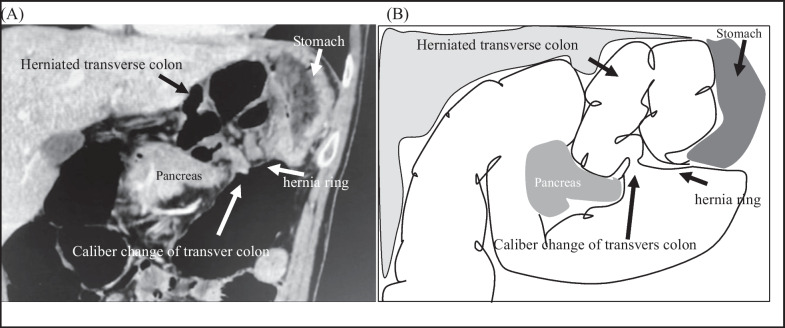
Preoperative contrast-enhanced computed tomography image, coronal view. **A** Note the change in the caliber of the transverse colon and the presence of a hernia ring in the lesser curvature of the stomach. The transverse colon has formed closed loop on the cephaloventral side of the stomach. **B** Schema of the image

**Fig. 2:**
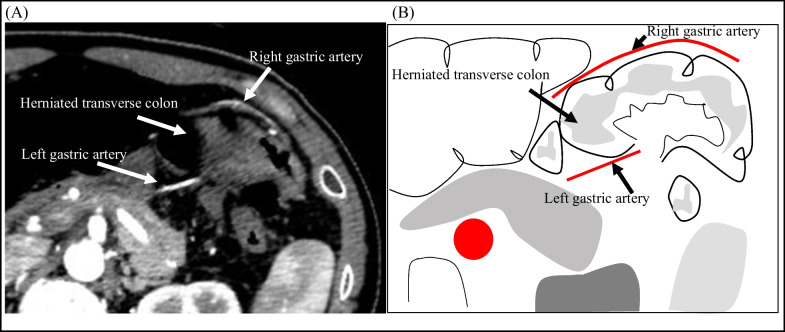
Preoperative contrast-enhanced computed tomography image, axial view. **A** The right gastric artery is ventral to the herniated transverse colon, and the left gastric artery is dorsal to the herniated transverse colon. **B** Schema of the image

**Fig. 3:**
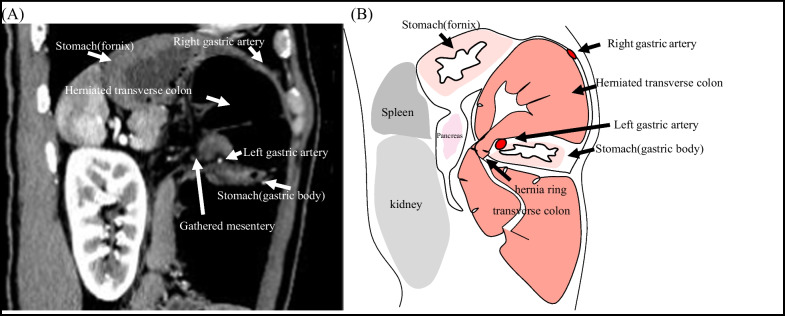
Preoperative contrast-enhanced computed tomography image, sagittal view. **A** The transverse colon was herniated and located ventral to the gastric body. The gastric body was compressed cephalocaudally by the dilated transverse colon. The mesentery gathered between the pancreas and the gastric body at the hernia ring. **B** Schema of the image

**Fig. 4:**
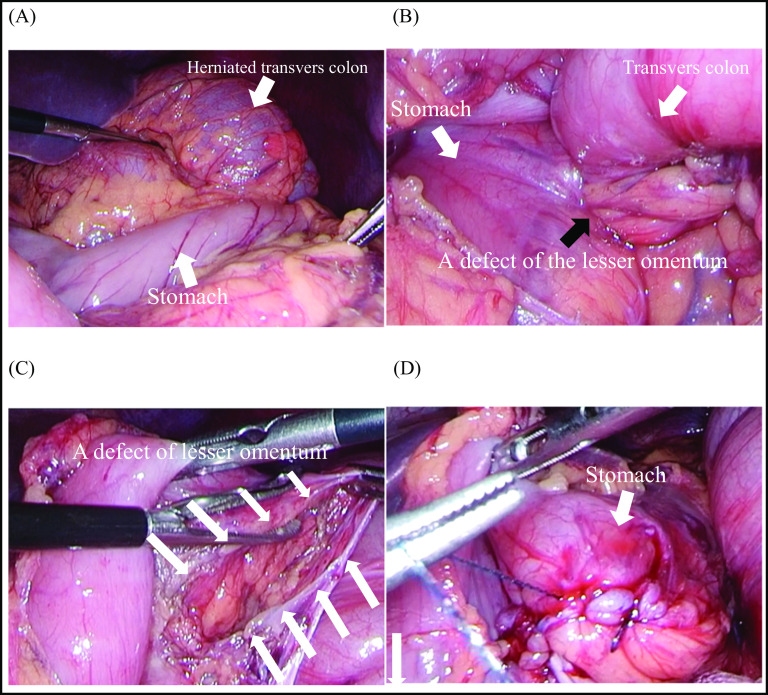
Laparoscopic findings. **A** The dilated transverse colon was located on the cephalic side of the stomach and was covered by a layer of lesser omentum. **B** The transverse through the defect of the lesser omentum (black arrow). **C** A defect of the posterior layer of the lesser omentum after widening the small defect with laparoscopic coagulation shears. **D** Sutured defect of the lesser omentum

A 43-year-old man was admitted to the emergency department with a 2-day history of acute abdominal pain. He presented with a history of an enlarged prostate but no history of abdominal surgery or trauma. At presentation, his blood pressure was 106/75 mmHg and his pulse rate was 83 bpm. His abdomen was mildly distended, and tenderness was noted throughout the abdomen. He had bilious vomiting in the emergency room. Laboratory tests were unremarkable, as expected for leukocytosis (12,000/mL). Contrast-enhanced computed tomography (CT) of the abdomen revealed a change in the caliber of the transverse colon and the presence of a hernia ring in the lesser curvature of the stomach. The transverse colon formed a closed loop on the cephaloventral side of the stomach (Fig. 1). On axial contrast-enhanced CT images, the right and left gastric arteries were ventral and dorsal to the herniated intestine, respectively (Fig. 2). As shown in the sagittal contrast-enhanced CT images, the transverse colon was herniated and located ventral to the gastric body. The gastric body was compressed cephalocaudally by the dilated transverse colon. The mesentery gathered between the pancreas and the gastric body at the hernia ring (Fig. 3). The patient was diagnosed with lesser omental hernia based on the above mentioned findings, and he underwent laparoscopic surgery the same day of diagnosis. Intraoperatively, the anterior layer of the lesser omentum covered the transverse colon (Fig. 4). The gastrocolic ligament was absent owing to the dysplasia of the greater omentum, and the bursa omental space was already free. The dorsal gastric corpus was lifted, which revealed a 2-cm defect in the posterior layer of the lesser omentum, and the transverse colon had formed an internal hernia. A 2-cm incision was made in the posterior layer of the lesser omentum with laparoscopic coagulation shears to widen the small defect. The herniated intestinal section was withdrawn from the hernia sac, and no ischemia was observed in the intestinal tract; therefore, the transverse colon was not resected. The hiatus of the lesser omentum was closed with continuous sutures. The patient was discharged on the fifth postoperative day.

## Discussion

**Fig. 5:**
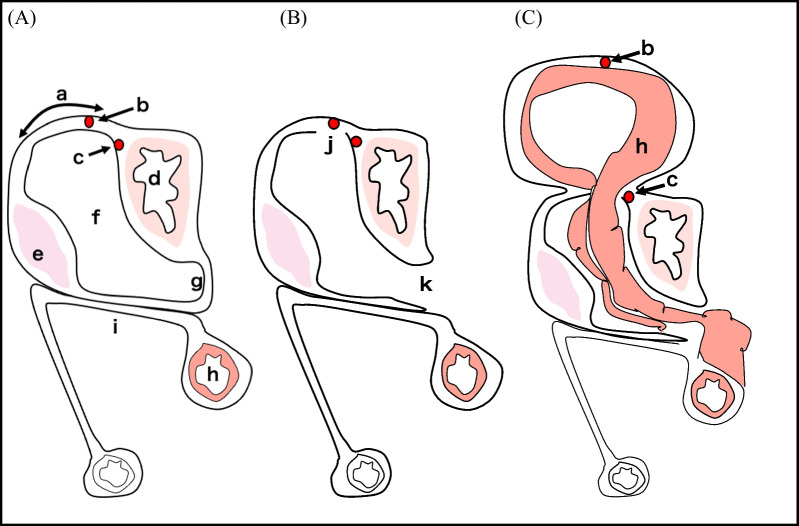
The schema of the lesser omental hernia in sagittal view. **A** Normal position of intra-abdominal organs and membrane anatomy. **B** The schema illustrating the position of intra-abdominal organs in the current patient. Note the defect in the posterior membrane of the lesser omentum (j) and dysplasia of the greater omentum (k). **C** The schema illustrating the hernia through the lesser omentum in the current. The transverse colon has passed through the defect (j) and herniated between the anterior and posterior layers of the lesser omentum. The herniated transverse colon is situated between the right and left gastric arteries. a, lesser omentum; b, right gastric artery; c, left gastric artery; d, stomach; e, pancreas; f, bursa omentalis; g, greater omentum; h, transverse colon; i, mesentery of transverse colon; j, hiatus of the posterior lesser omentum; k, dysplasia of the greater omentum

Although lesser omental hernias are more common after colostomies [1, 5, 11], several studies reported lesser omental hernias in patients without a history of abdominal surgery [6–10]. In all previously reported cases, intestinal herniation occurred through both layers of the lesser omentum [1, 3–11]. In contrast, in the present case, the internal hernia occurred through a defect that included only the posterior layer of the lesser omentum (Fig. 5).

Gastric nutrient vessels, such as the right and left gastric arteries, reside between the anterior and posterior layers of the lesser omentum. In the present case, the transverse colon entered through a defect in the posterior layer without penetrating the anterior layer to herniate between the anterior and posterior layers. During laparoscopy, the transverse colon was covered by the anterior layer, and the defect was observed in the posterior layer of the lesser omentum on the dorsal side of the stomach.

Previous studies reported internal hernia due to a defect in the broad ligament of the uterus, which is also a double-layered peritoneal structure [12, 13]. Internal hernia involving the broad ligament of the uterus can be classified into two types [14]. The fenestra type involves a complete opening in the broad ligament, whereas the pouch type involves a defect in the anterior or posterior layer. Notably, the intestine becomes herniated between the anterior and posterior layers. The pouch type is rarer than the fenestra type. Although pouch-type hernia of the lesser omentum can also occur, to date, only the fenestra-type hernias of the lesser omentum have been reported. To the best of our knowledge, this is the first report of a pouch-type hernia of the lesser omentum.

The diagnosis of lesser omental hernia is difficult because of the nonspecific findings in physical examination and blood tests [1], and CT findings are crucial for making a definitive diagnosis. The CT findings of lesser omental hernia include dilated bowel loops located in the ventral part of the stomach, mesentery gathered in the lesser curvature of the stomach where the hernia ring is also present, and distorted and relocated stomach, with similar changes that may occur in surrounding organs [5]. The locations and correlation between the herniated intestinal tract, stomach, mesentery, and hernia ring can be distinguished on the sagittal CT image (Fig. 3). Contrast-enhanced CT imaging of the present case revealed two characteristic findings: compressed and thinning of the gastric body (Fig. 3) and the herniation of the transverse colon between the contrast-enhanced right and left gastric arteries (Fig. 2).

Herniated bowel of fenestra-type lesser omental hernias occupies free space in the intra-abdominal cavity. However, herniated bowel of a pouch-type lesser omental hernia occupies the closed space of the lesser omentum. Therefore, the dilated herniated bowel in our case was next to the stomach and compressed the stomach from the head side. The dilated transverse colon, oral to the hernia ring, compressed the stomach from the caudal side. Finally, the sagittal CT image showed thinning of the gastric body (Fig. 3). This finding is characteristic of the posterior layer of a lesser omental hernia. The presence of the intestinal tract between the right and left gastric arteries, which originally travel within the lesser omentum, indicated that the hernia was located between the anterior and posterior layers of the lesser omentum. We refer to this CT finding as a sandwich sign. The presence of this sign on contrast-enhanced CT may aid in the definitive diagnosis of hernia forming between the anterior and posterior layers of the lesser omentum.

In the present case, surgery was successfully completed with the laparoscopic approach. Attention was paid to prevent damage to the left gastric artery during the incision of the posterior layer of the lesser omentum with the ultrasonic coagulating incision device. In the previous reports of lesser omental hernias, laparotomy was the most commonly chosen procedure, and laparoscopic surgery was used in only two cases [1, 11]. Considering the use of minimally invasive procedures, laparoscopic surgery may be a viable option for these patients.

## Conclusions

As illustrated in the present patient, who presented with hernia forming between the anterior and posterior layers of the lesser omentum, characteristic CT findings may play an active role in the diagnosis of this rare presentation.

## Data Availability

The datasets used and/or analyzed during the current study are available from the corresponding author on reasonable request.

## Abbreviation

CT: Computed tomography
